# Clonal diversity and genomic characterization of Panton-valentine Leukocidin (PVL)-positive *Staphylococcus aureus* in Tehran, Iran

**DOI:** 10.1186/s12879-021-06060-4

**Published:** 2021-04-21

**Authors:** Zahra Najafi olya, Shahin Najar-Peerayeh, Abbas Yadegar, Bita Bakhshi

**Affiliations:** 1 Department of Bacteriology, Faculty of Medical Sciences, Tarbiat Modares University, Jalal-Ale-Ahmad Ave, Tehran, 14117-13116 Iran; 2 Foodborne and Waterborne Diseases Research Center, Research Institute for Gastroenterology and Liver Diseases, Shahid Beheshti University of Medical Sciences, Tehran, Iran

**Keywords:** *Staphylococcus aureus*, Panton-valentine leukocidin, Haplotype groups, MLST, PFGE, Iran

## Abstract

**Background:**

Some *Staphylococcus aureus* strains produce Panton-Valentine leukocidin (PVL), a bi-component pore-forming toxin, which causes leukocyte lysis and tissue necrosis. Currently, there is very limited information on the molecular epidemiology of PVL-encoding *S. aureus* strains in Iran. This study aimed to determine the molecular epidemiology and genetic background of PVL-positive *S. aureus* clinical strains isolated from Iranian patients.

**Methods:**

A total of 28 PVL-positive *S. aureus* strains were detected from 600 *S. aureus* isolates between February 2015 and March 2018 from different hospitals in Tehran, Iran. Antimicrobial susceptibility testing was performed according to the Clinical and Laboratory Standards Institute (CLSI) guidelines. Molecular genotyping was performed using SCC*mec* and accessory gene regulator (*agr*) typing, PVL haplotyping, multilocus sequence typing (MLST), and pulsed-field gel electrophoresis (PFGE).

**Results:**

The highest antibiotic resistance rate was found to be against erythromycin (57.1%), followed by ciprofloxacin (42.8%) and clindamycin (35.7%). Moreover, 19 (67.9%) out of 28 *S. aureus* isolates were identified as MRSA, including CA-MRSA (14/19, 73.7%) and HA-MRSA (5/19, 26.3%). SCC*mec* type IVa was detected as the predominant type (10/19, 52.6%), followed by type III (5/19, 26.3%) and type V (4/19, 21.1%). The *agr* type I was identified as the most common type (14/28, 50%), and H and R haplotype groups were observed at frequencies of 67.9 and 32.1%, respectively. Among H variants, the predominant variant was H2 (78/9%). The isolates encompassed 21 different sequence types (STs), including 16 new STs (ST5147 to ST5162). Based on eBURST analysis, the isolates were clustered into five CCs, including CC30, CC22, CC1, CC8, and CC5 (ST5160), and nine singletons. PFGE typing showed that 24 isolates were clustered into A (4 pulsotypes), B (9 pulsotypes), and C (11 pulsotypes) clusters.

**Conclusions:**

A high prevalence of PVL-positive CA-MRSA strains was detected in Iran. The majority of PVL-positive isolates were of H (mostly H2) variant, while R variant was harbored by 100% of PVL-positive MRSA strains. Also, CC8, CC22, and CC30 were identified as the dominant clones among PVL-encoding *S. aureus* strains. This study promotes a better understanding of the molecular epidemiology and evolution of PVL-positive *S. aureus* strains in Iran.

## Background

*Staphylococcus aureus* (*S. aureus*)-associated disorders vary from skin infections to life-threatening invasive diseases, such as bacteremia, sepsis, and endocarditis, mediated by a variety of virulence factors [1, 2]. *S. aureus* plays an important role in the development of epidural abscesses, meningitis, toxic shock syndrome (TSS), urinary tract infections (UTIs), septic thrombophlebitis, pneumonia, etc. [3, 4]. *S. aureus* causes invasive infections in all age groups, but the prevalence of these infections is somewhat higher in infants and patients over 65 years of age [3]. Panton-Valentine Leukocidin (PVL) is a two-component toxin produced by some *S. aureus* strains in varying amounts [5, 6]. However, the majority of isolates that cause skin and soft tissue infections (SSTI) and severe necrotizing pneumonia are PVL-positive [7]. This preforming toxin is encoded by a 1.9-kb *lukSF-PV* locus consisting of two contiguous, but co-transcribed *lukF* and *lukS* genes [8]. It is well documented that these two components are secreted by *S. aureus* strains, and before they assemble into a pore-forming heptamer on neutrophil membranes, they could induce lysis of host defense cells, including human polymorphonuclear neutrophils (PMNs), monocytes, and macrophages [9, 10]. PVL causes apoptosis in neutrophils through the activation of caspase-3 and -9; the participation of TLR2 (toll like receptor 2) in causing inflammation by PVL in the lung has also been reported [4].

Generally, phages are considered as one of the major mobile genetic elements (MGEs) among *S. aureus* strains, which are strongly able to transfer antibiotic resistance markers and virulence attributes [11, 12]. The genes encoding PVL are also located on lysogenized bacteriophages integrated into the chromosomal content of *S. aureus* [9]. Currently, these phages are classified into the order Caudovirales, which could be divided into three major families based on the tail morphology, including Podoviridae, Siphoviridae, and Myoviridae [8]. To date, at least 10 PVL phages belonging to the Siphoviridae family have been identified and sequenced, including 108PVL, PVL, tp310–1, SLT, Sa2958, Sa2MW, Sa2usa, 7247PVL/ST5967PVL, TCH60, and Sa119 PVL [13–17]. They are characterized as double-stranded, non-enveloped DNA viruses with an icosahedral head and a long non-contractile tail [5, 12].

The emergence of PVL-positive methicillin-resistant S. *aureus* (PVL-MRSA) isolates have been reported worldwide [18]. Previous studies have revealed a strongassociation between the presence of PVL genes and community-associated MRSA (CA-MRSA) strains, especially those carrying staphylococcal cassette chromosome*mec* (*SCCmec*) types IV [19]. Moreover, hospital-acquired MRSA (HA-MRSA) strains that carry PVL genes have been reported in various geographical regions inEurope and Asia [9, 19, 20]. Generally, infections caused by PVL-positive methicillin-sensitive S. *aureus* (PVL-MSSA) strains have been reported to play the role ofreservoirs for PVL-MRSA due to clonality and evolutionary relationships [21].

Some studies have shown an association between PVL genes and invasive diseases, implying that PVL is an epidemiological marker for severe infection syndrome; also, individuals with PVL-positive skin and soft-tissue infections are more likely to require surgery compared to those with PVL-negative infection. In some countries, this notion has led to the implementation of public health measures for individuals infected with PVL-producing strains. Compared with PVL-negative *S. aureus*, PVL-positive *S. aureus* strains are more likely to be truly community-acquired, infecting individuals who have not had contact with healthcare settings [10]. However, the potential risk of spreading PVL-positive *S. aureus* strains to hospitals is considered as a significant public health concern, as the establishment of a PVL-positive clonal lineage of HA-MRSA strains could rapidly lead to dramatically worse outcomes for HA-MRSA patients. However, there are clonal lineages from which HA-MRSA and CA-MRSA have been reported [5].

To date, at least 22 single-nucleotide polymorphisms (SNPs) have been identified in the *lukSF-PV* genes based on phylogenetic analysis [8, 13, 22]. Additionally, a number of non-synonymous mutations have been detected in different isoforms of PVL protein. PVL-positive *S. aureus* strains could be classified into four major haplotype groups (R, H1, H2, H3) based on non-synonymous variations in the PVL sequence at nucleotide positions 527, 663, and 1396 [22, 23].

The population structure and clones of MRSA strains are changing in different healthcare facilities in different countries. The most prevalent PVL-MRSA types in the United States belong to ST8 (USA300), ST1 (USA400), ST59 (USA1000), and ST30 (USA1100), while ST80 clone is commonly reported in European countries [24]. Furthermore, several clones belonging to ST80, ST30, ST59, and ST22 have been reported as the most frequent clones in Asia [24–26]. Previous studies in Iran have reported ST22, ST30, ST8, ST931, ST722, ST15, ST88, ST239, ST291, and ST585 as the predominant clones among MRSA strains [27–29]

Currently, there is very limited data on the molecular epidemiology of PVL-encoding *S. aureus* clinical strains in Iran. In addition, no details have yet been reported about PVL haplotype groups of *S. aureus* strains in Iran. The present study aimed to obtain a more complete description about the molecular epidemiology and genetic background of PVL-positive *S. aureus* clinical strains isolated from Iranian patients using a combination of molecular typing techniques, including SCC*mec* and accessory gene regulator (*agr*) typing, PVL haplotyping, multilocus sequence typing (MLST) analysis, and pulsed-field gel electrophoresis (PFGE). The antibiotic susceptibility of the strains was also determined.

## Materials and methods

### Bacterial strains

In this study 600 *S. aureus* isolates were analysed and a total of 28 PVL-positive *S. aureus* strains were collected from February 2015 to March 2018 from different hospitals in Tehran, Iran. The strains were obtained from both outpatients (24 strains) and inpatients (4 strains). Clinical data and demographic information were recorded for all patients enrolled in this study using a questionnaire. All patients or their legal guardians provided their written informed consent. This study was approved by the Medical Ethics Committee of Tarbiat Modares University before it began. In addition, all methods were carried out in accordance with relevant guidelines and regulations at Tarbiat Modares University.

All *S. aureus* strains were identified using conventional phenotypic and biochemical examinations, including colony morphology, Gram staining, mannitol fermentation on mannitol salt agar (MSA), positive reactions in catalase, slide and tube coagulase, and DNase tests. Isolates were preserved in tryptic soy broth (TSB) with 20% glycerol (v/v) at − 80 °C.

### Antimicrobial susceptibility testing

Antimicrobial susceptibility testing was performed by the Kirby-Bauer disk diffusion method according to the Clinical and Laboratory Standards Institute guidelines [31] using the following antibiotics (Mast Diagnostics, UK): chloramphenicol (30 μg), amikacin (30 μg), erythromycin (15 μg), clindamycin (2 μg), gentamicin (10 μg), linezolid (30 μg), ciprofloxacin (5 μg), trimethoprim-sulfamethoxazole (25 μg), rifampicin (5 μg), and cefoxitin (30 μg). *S. aureus* ATCC 25923 was used for quality control.

### Genomic DNA preparation and detection of *pvl* gene

Genomic DNA was extracted using Gram Positive DNA Purification Kit (Gene Transfer Pishgaman, Iran) according to the manufacturer’s protocol. DNA samples were evaluated in terms of quality by electrophoresis on 0.8% (w/v) agarose gels and then stored at − 20 °C until used for PCR analysis. PCR was used to confirm the presence of *pvl* gene in all strains using specific primers (pvl-F GGAAACATTTATTCTGGCTATAC and pvl-R CTGGATTGAAGTTACCTCTGG), yielding a 502 bp fragment as previously described [32].

### Detection of MRSA strains and SCC*mec* typing

All *S. aureus* strains in this study were screened for MRSA according to resistance to cefoxitin (30 μg) using the Kirby-Bauer disk diffusion method ([33]). The identity of MRSA strains was confirmed by detection of *mec*A using PCR method ([33]). A multiplex PCR-based method was also carried out for SCC*mec* typing as previously described [32]. The oligonucleotide primers used to amplify the genes of interest are listed in Table 1.

**Table 1:** Oligonucleotide primers used in this study

Primer	Sequence (5′ → 3′)	Product size (bp)
SCC*mec* I	F: GCTTTAAAGAGTGTCGTTACAGG R: GTTCTCTCATAGTATGACGTCC	613
SCC*mec* II	F: CGTTGAAGATGATGAAGCG R: CGAAATCAATGGTTAATGGACC	398
SCC*mec* III	F: CCATATTGTGTACGATGCG R: CCTTAGTTGTCGTAACAGATCG	280
SCC*mec* IVa	F: GCCTTATTCGAAGAAACCG R: CTACTCTTCTGAAAAGCGTCG	776
SCC*mec* IVb	F: TCTGGAATTACTTCAGCTGC R: AAACAATATTGCTCTCCCTC	493
SCC*mec* IVc	F: ACAATATTTGTATTATCGGAGAGC R: TTGGTATGAGGTATTGCTGG	200
SCC*mec* IVd	F: CTCAAAATACGGACCCCAATACA R: TGCTCCAGTAATTGCTAAAG	881
SCC*mec* V	F: GAACATTGTTACTTAAATGAGCG R: TGAAAGTTGTACCCTTGACACC	325
PanF	F: ATGCACATGGTGCACATGC	
*agr* I	R: GTCACAAGTACTATAAGCTGCGAT	441
*agr* II	R: TATTACTAATTGAAAAGTGGCCATAGC	575
*agr* III	R: GTAATGTAATAGCTTGTATAATAATACCCAG	323
*agr* IV	R: CGATAATGCCGTAATACCCG	659

### *agr* genotyping by multiplex PCR

The *agr* types (I–IV) were determined by a multiplex PCR assay as described by Shopsin et al. [34]. The oligonucleotide primers used to amplify the genes of interest are listed in Table 1.

### Determination of PVL haplotype groups

A PCR-based sequencing method was applied to determine SNPs in the *lukSF-PV* genes of all *S. aureus* strains. PCR amplifications were performed using a primer pair (lukS-F GTGGTCCATCAACAGGAGGT and lukF-R TGGTCCCCAACCATTATTCA) specifically designed to generate a 1107 bp fragment (nucleotides 440 to 1546) of the *lukSF-PV* genes. PCR was carried out in a final volume of 25 μL reaction mixture containing 10 μL of Taq DNA Polymerase Master Mix (Ampliqon, Denmark), 0.5 μL of each primer (10 pmol), and 2 μL of template DNA (approximately 200 ng) using a thermocycler (Eppendorf, Hamburg, Germany). Amplifications were run under the following cycling conditions: an initial denaturation at 94 °C for 5 min; followed by 30 cycles of denaturation at 94 °C for 1 min, 57 °C for 1 min, and 72 °C for 1 min; and a final elongation step at 72 °C for 5 min. Sanger sequencing of both strands was performed using an automated sequencer (Microsynth, Balgach, Switzerland). DNA sequences were edited by Chromas Lite software Version 2.5.1 (Technelysium Pty Ltd., Australia). The edited nucleotide sequences of *LukSF-PV* genes were subjected to in-frame translation using BioEdit software Version 7.2.5 and aligned to the sequence of MRSA strain USA300 (GenBank: CP000255.1) as a reference sequence.

### MLST

MLST was performed for all *S. aureus* strains using previously reported primers specific for seven housekeeping genes, including *arcC*, *aroE*, *glpF*, *gmk*, *pta*, *tpi*, and *yqiL,* according to the previously described protocol [35]. The sequences of the PCR products were compared to those of the existing alleles available on the MLST website (https://pubmlst.org/saureus/) and analyzed online to assign allelic profile (sequence type, ST) and the associated clonal complex (CC). The minimum spanning tree (MST) was constructed by the goeBURST algorithm using PHYLOViZ 2.0 software (https://www.phyloviz.net/).

### PFGE

PFGE was performed using *Sma*I restriction enzyme (Takara, Japan) digestion according to the protocol described by the Center for Disease Control and Prevention (CDC) with minor modifications (https://www.cdc.gov/pulsenet/pathogens/pfge.html). The chromosomal DNA of *Salmonella enterica* serovar Braenderup (H9182) was used as the normalization standard and molecular marker. Digested plugs were loaded into the wells of a 1% agarose gel and run in 0.5X TBE using a CHEF (contour-clamped homogeneous electric field)-DR III system (Bio-Rad, Hercules, CA). After completing the electrophoresis process, the gel was stained using a 1.5 μg/mL ethidium bromide solution for 30–40 min on a rocking shaker in a covered container. Destaining was done three times with distilled water on the shaker for 45 min, and then the gel was visualized and photographed. Gel photos were processed and analyzed using GelCompare II software V. 4.0 (Applied Maths, Saint Martens-Latem, Belgium). Similarities between electrophoretic patterns were calculated using the Dice coefficient and set to 80% to determine the pulsed-field type clusters after reviewing the epidemiologic data associated with each of the clusters of isolates. The unweighted pair group method with arithmetic means (UPGMA) was used to construct a dendrogram with 1.5% tolerance and optimization as standard settings.

## Results

### Antibiotic resistance profiles

**Fig. 1:**
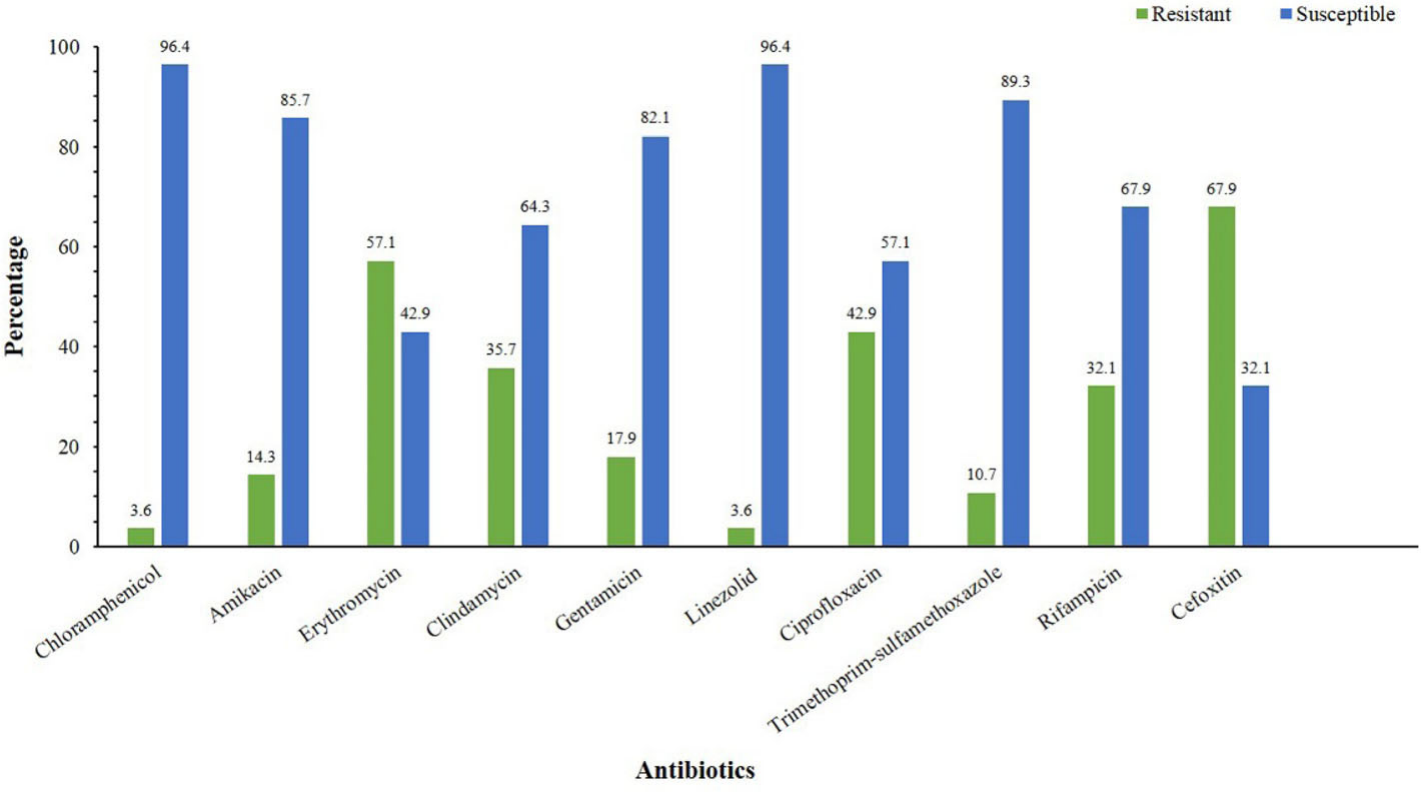
Bar graph presenting antimicrobial susceptibility patterns of 28 PVL-positive *S. aureus* isolates in this study

The antibiotic resistance profiles of 28 PVL-positive *S. aureus* isolates against various antibiotics are shown in Fig. 1. The highest resistance was observed against erythromycin (16/28, 57.1%), followed by ciprofloxacin (12/28, 42.8%), clindamycin (10/28, 35.7%), rifampicin (9/28, 32.1%), gentamicin (5/28, 17.8%), amikacin (4/28, 14.3%), and trimethoprim-sulfamethoxazole (3/28, 10.7%). The lowest resistance was observed against linezolid and chloramphenicol with the same rate (1/28, 3.6%). Of 28 PVL-positive *S. aureus* isolates, 19 (67.9%) isolates were MRSA, including CA-MRSA (14/19, 73.7%) and HA-MRSA (5/19, 26.3%). Among MRSA isolates, the highest resistance was observed against erythromycin (10/19, 52.6%) and ciprofloxacin (8/19, 42.1%). All MRSA isolates were susceptible to linezolid, and resistance to trimethoprim-sulfamethoxazole and chloramphenicol was observed in 10.5 and 5.3% of the isolates, respectively. Of 19 MRSA isolates, 5 (26.3%) were found to be resistant to at least three tested antibiotics. Among MSSA (9/28, 32.1%) isolates, the highest resistance rate was observed against erythromycin and clindamycin (6/9, 66.7%). All MSSA isolates were found to be susceptible to chloramphenicol and amikacin.

### SCC*mec* and *agr* typing

By SCC*mec* typing, only three types (types III, IVa, and V) were found among 19 PVL-positive MRSA isolates. The most common type of SCC*mec* was type IVa (10/19, 52.6%), followed by type III (5/19, 26.3%) and type V (4/19, 21.1%).

The *agr* types of all 28 PVL-positive *S. aureus* isolates (MSSA and MRSA) indicated that *agr* type I was the predominant one (14/28, 50%), followed by type III (10/28, 35.8%), type II (3/28, 10.7%), and type IV (1/28, 3.5%). The detailed antibiotic resistance profile and molecular characterization of PVL-positive isolates by SCC*mec*, *agr*, haplotype, MLST, and PFGE typing are shown in Table 2.

**Table 2:** The detailed antibiotic resistance profile and molecular characterization of 28 PVL-positive *S. aureus* isolates by haplotype, *agr*, SCC*mec*, MLST and PFGE typing

Haplotype group	***agr*** type	SCC***mec***	ST	CC	PFGE	Antibiotic resistance	***lukS-PV*** GenBank	***lukF-PV*** GenBank
R	I	III	ST30	CC30	C	AK, E, CD, GM, CIP, RI, FOX	MT468511	MT468539
	II	V	ST30	CC30	C	E, CD, CIP, RI, FOX	MT468512	MT468540
	I	V	ST30	CC30	C	E, CIP, FOX	MT468514	MT468542
	III	IV	ST30	CC30	NT	E, CIP, FOX	MT468515	MT468543
	I	IV	ST22	CC22	C	FOX	MT468520	MT468548
	III	IV	ST1136	CC1	C	E, FOX	MT468517	MT468545
	III	IV	ST1136	CC1	C	E, FOX	MT468518	MT468546
	I	IV	ST1996	Singleton	C	FOX	MT468510	MT468538
	I	IV	ST121	Singleton	C	CIP, FOX	MT468519	MT468547
H1	I	–	ST5148	CC30	B	CD, LZD, RI	MT468494	MT468522
	IV	III	ST5153	CC22	A	E, CD, GM, CIP, RI, FOX	MT468499	MT468527
	I	–	ST5147	CC8	B	AK, E, CD, GM, CIP, SXT, RI	MT468493	MT468521
	I	IV	ST5151	Singleton	NT	FOX	MT468497	MT468525
H2	III	V	ST30	CC30	NT	SXT, FOX	MT468513	MT468541
	III	IV	ST30	CC30	B	E, FOX	MT468516	MT468544
	III	III	ST5150	CC22	C	C, E, CIP, FOX	MT468496	MT468524
	I	–	ST5155	CC22	B	E, CD	MT468501	MT468529
	III	III	ST5157	CC22	A	AK, GM	MT468503	MT468531
	I	III	ST5156	CC8	B	AK, E, CD, GM, CIP, RI, SXT, FOX	MT468502	MT468530
	III	IV	ST5158	CC8	C	FOX	MT468504	MT468532
	I	–	ST5149	CC1	B	E, CD, CIP, RI	MT468495	MT468523
	II	–	ST5160	CC5	B	E, CD	MT468506	MT468534
	III	–	ST5152	Singleton	B	E, CD	MT468498	MT468526
	III	–	ST5154	Singleton	C	CIP, RI	MT468500	MT468528
	I	IV	ST5159	Singleton	A	FOX	MT468505	MT468533
	I	V	ST5161	Singleton	NT	FOX	MT468507	MT468535
	I	–	ST5162	Singleton	B	E, CD, CIP, RI	MT468508	MT468536
	II	–	ST1996	Singleton	A	NR	MT468509	MT468537

*-* no SCC*mec* elements were detected (MSSA)

*NT* non-typeable

*NR* non-resistant

### Haplotype groups

**Fig. 2:**
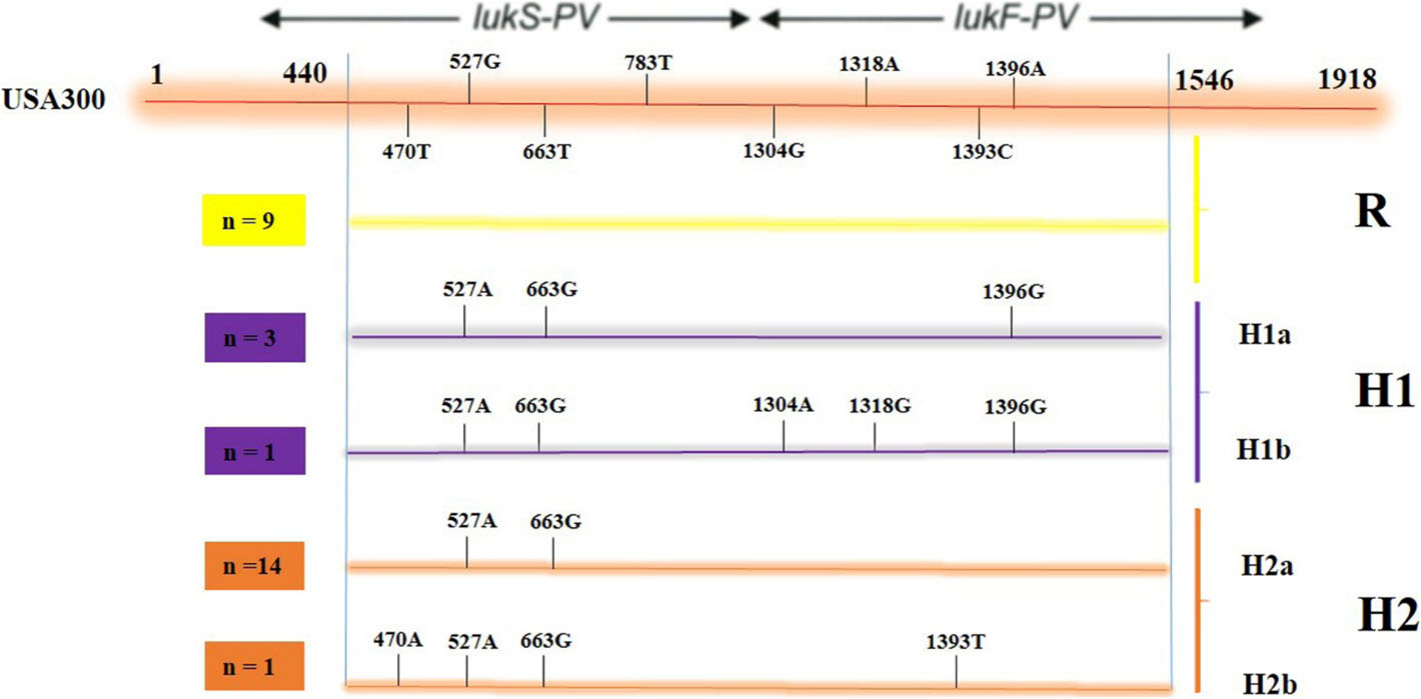
Schematic structure of the *lukSF-PV* sequence variants detected among 28 PVL-positive *S. aureus* strains in this study compared to the *lukSF-PV* sequence of USA300 as the reference strain. Red horizontal line indicates the *lukSF-PV* sequence of USA300 strain. Short vertical lines on the colored lines indicate the positions of sites at which the haplotype differs from that of USA300. Numbers to the left of the horizontal lines indicate the frequency with which the haplotype (R, H1, and H2) was observed. At right, the haplotypes are bracketed according to how they are grouped into haplotype groups

Approximately 1107 bp fragments of *lukSF-PV* genes of 28 PVL-positive *S. aureus* isolates were amplified by PCR, and the products were sequenced. As expected, the sequences were highly conserved, but nucleotide variations were observed at seven sites (positions 470, 527, and 663 located in the *lukS* locus and positions 1304, 1318, 1393, and 1396 located in the *lukF* locus) using the *lukSF-PV* genes of MRSA strain USA300 as a reference. Among the isolates, 19 (67.9%) isolates were of H variant, representing the most common types as defined by O’Hara et al. [22], and the remaining nine (32.1%) isolates were identified as R variant, displaying non-synonymous mutation at nucleotide 527 A to G. Also, H variants were further classified into H1 (4/28, 14.3%) and H2 (15/28, 53.6%) groups which differed in nucleotide position 1396. Furthermore, H1 variants were further divided into H1a (*n* = 3) and H1b (*n* = 1) groups according to the differences in nucleotide sites 1304 and 1318. Additionally, H2 variants were grouped into H2a (*n* = 14) and H2b (*n* = 1) groups according to the difference in nucleotide site 470. The schematic structure of the *lukSF-PV* sequence variants found in 28 PVL-positive *S. aureus* clinical strains under study compared to the *lukSF-PV* sequence of USA300 strain is shown in Fig. 2. All R variants were MRSA, but only 50% of H1 variants were MRSA. Both R and H1 variants were isolated from the wound and hospitalized patients, except for one case in each variant. H2 variants were isolated from wound, blood and tracheal cultures and 53.3% were MRSA.

### MLST

The evolutionary and genetic diversity of 28 PVL-positive *S. aureus* isolates was analyzed by MLST (Table 2). In this study, 21 distinct STs were identified among the isolates, of which 16 STs did not have matching profiles in the MLST database and were subsequently designated to ST5147-ST5162 after submitting the data to the website (https://pubmlst.org/saureus/). Among the obtained STs, the most frequently identified one was ST30 (6/28, 21.4%), followed by ST1996 and ST1136, each accounted for two isolates (2/28, 7.1%). Other STs accounted for one isolate. ST30 was the predominant type (6/19, 31.6%) among MRSA isolates, while no predominant ST was found among MSSA isolates.

**Fig. 3:**
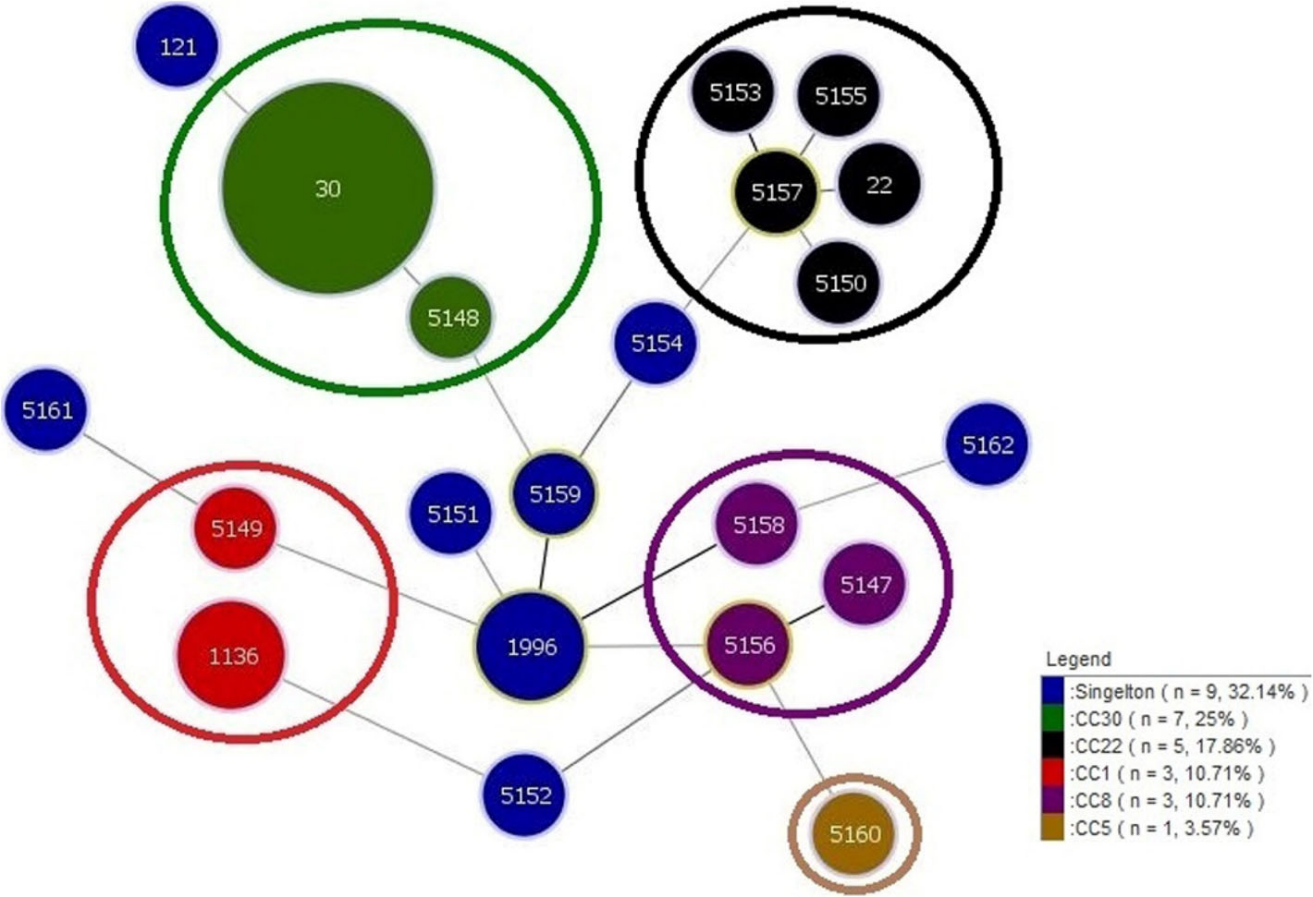
The minimum spanning tree (MST) of 28 *S. aureus* strains was constructed by the goeBURST algorithm using the PHYLOViZ 2.0 software (https://www.phyloviz.net/). Each circle represents a unique sequence type (ST). The size of each circle is proportional to the numbers of isolates per ST. The circles are color-coded according the clonal complex (CC) and singletons. The length between two nodes reflects the genetic distance between the two bordering STs. Similar CCs have color-coded in the legend

**Fig. 4:**
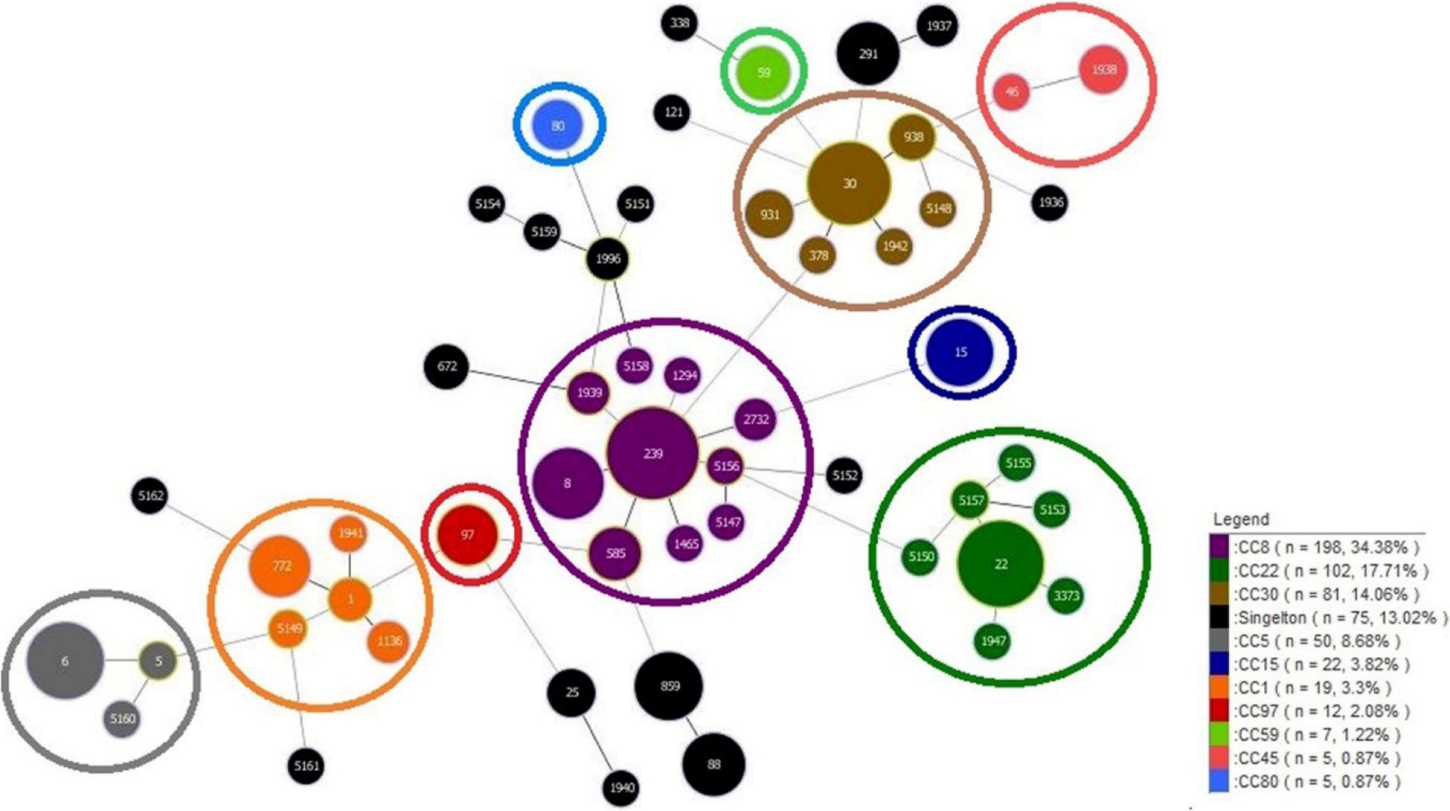
The minimum spanning tree (MST) of 576 Iranian *S. aureus* strains was constructed by the goeBURST algorithm using the PHYLOViZ 2.0 software (https://www.phyloviz.net/). The allelic profiles were downloaded from the MLST website (https://pubmlst.org/saureus/) (105 STs) and previously described in the literature (443 STs), which included the sequence types (STs), clonal complexes (CCs) and singletons as well as 28 STs in this study. Each circle represents a unique ST. The size of each circle is proportional to the numbers of isolates per ST. The circles are color-coded according the CCs and singletons. The length between two nodes reflects the genetic distance between the two bordering STs. Similar CCs have color-coded in the legend

Based on eBURST analysis and by using all *S. aureus* STs available in the MLST database, the isolates were clustered into five CCs, including CC30 (ST30, ST5148), CC22 (ST22, ST5152, ST5153, ST5155, ST5157), CC1 (ST1136, ST5149), CC8 (ST5148, ST5156, ST5158), and CC5 (ST5160), and nine singletons (ST121, ST1996, ST5151, ST5152, ST5154, ST5159, ST5161, ST5162). The largest cluster was CC30 with seven isolates (25%), followed by CC22 with five isolates (17.9%) as well as CC1 and CC8, each with three isolates (10.7%). The remaining CC5 included one isolate. Among MRSA isolates, the predominant cluster was CC30 including 31.6% (6/19) of the isolates, while no predominant CC was detected among MSSA isolates. Figures 3 and 4 represent the MLST of 576 *S. aureus* clones downloaded from the MLST website (105 STs) and 443 STs previously described in the literature [27–30, 36–42], as well as 28 STs identified in this study, respectively.

### PFGE

**Fig. 5:**
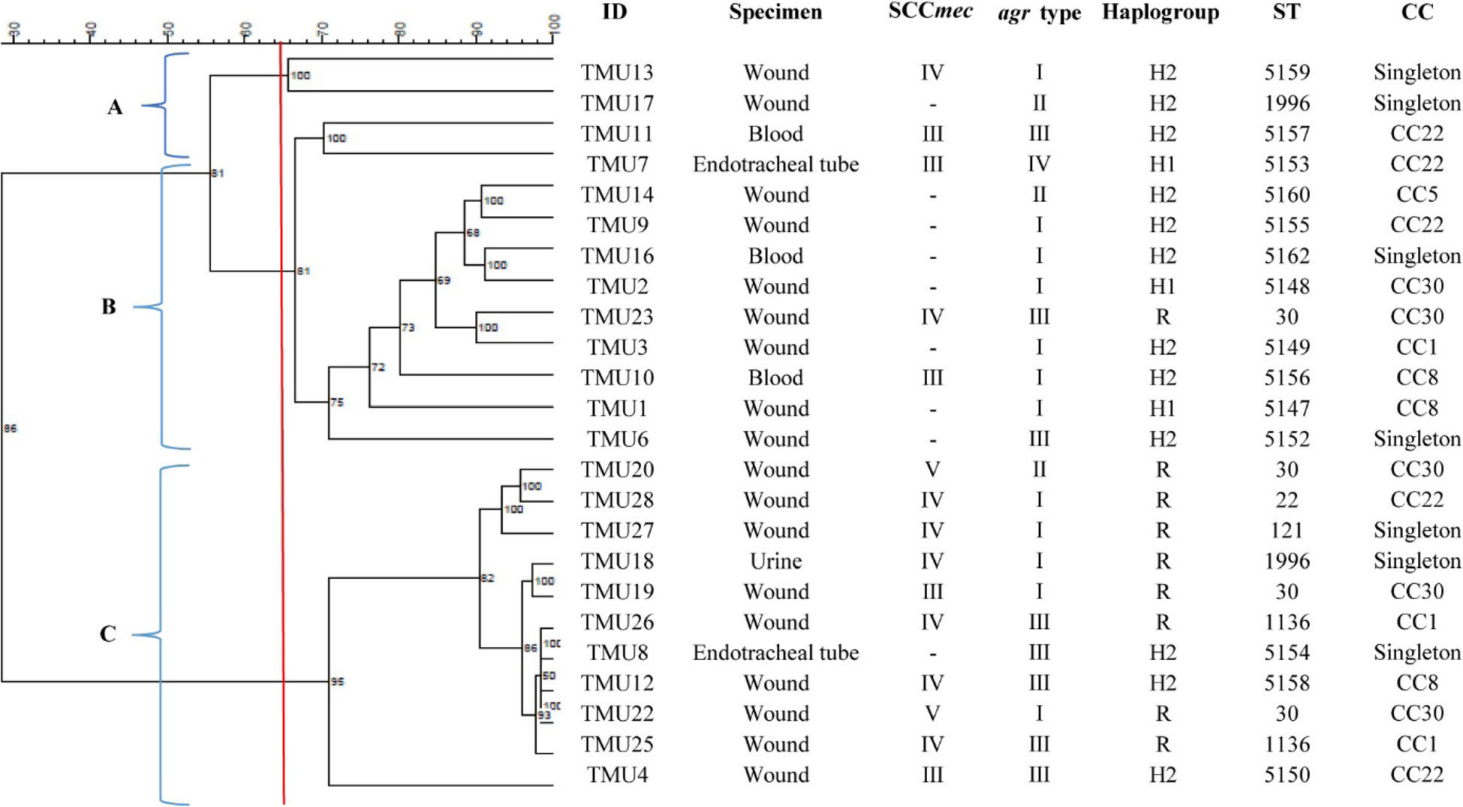
Dendrogram derived from UPGMA cluster analysis of the PFGE patterns and characteristics related to the genetic background of 24 PVL-positive *S. aureus* isolates. Isolates showing a similarity coefficient ≥ 65% were considered genetically related

The genetic relationship between 28 PVL-positive *S. aureus* isolates was determined by PFGE. PFGE typing showed that 24 isolates were clustered into three PFGE clusters (A, B, and C), and the remaining four isolates did not represent satisfactory results for typing. Most of the isolates were divided into PFGE cluster B (9 pulsotypes) and cluster C (11 pulsotypes), together accounting for 62.5% (15/24) of the isolates. The majority of MRSA isolates (10/15, 66.7%) and also R variants (8/9, 88.9%) were grouped in PFGE cluster C. Additionally, most of the H2 variants (7/12, 58.3%) were grouped in PFGE cluster B. Dendrogram depicting the PFGE patterns and characteristics related to genetic background of 24 PVL-positive *S. aureus* isolates is shown in Fig. 5.

## Discussion

MALDI-TOF MS-based DOT-MGA is also able to perform antimicrobial susceptibility testing within few hours, through specific novel peaks, but is applicable to only some MRSA strains [43]. Therefore, MRSA strains were detected and confirmed by targeting the *mec*A gene responsible for resistance in staphylococci species, located on mobile genetic element called SCCmec.

Little information is available about the predominant STs, CCs, and particularly haplotype groups of PVL-positive *S. aureus* strains in Iran. In the current study, attempts were made to raise awareness about the molecular relatedness and epidemiology of PVL-positive *S. aureus* strains among Iranian patients by applying a number of molecular typing techniques in this setting. In this study, the majority of the isolates (67.85%) were detected to be PVL-positive MRSA. This result is consistent with several previous reports with respect to high prevalence of PVL-positive strains recovered from CA-MRSA [44, 45]. Also, previous studies in Iran have reported a high prevalence of PVL-positive strains among MRSA isolates, ranging from 12 to 60% [27, 28, 40, 42, 46].

Epidemiological studies have revealed that PVL gene is commonly carried by CA-MRSA having SCC *mec* type IV [47]. The majority of MRSA strains in the current (50%) and previous studies in Iran carried SCC*mec* IV [30, 34, 36, 38, 41, 46, 48], supporting the hypothesize that SCC*mec* IV is probably more mobile than other SCC*mec* types. This hypothesis is further supported by the fact that most public health-associated MRSA infections reported in Iran are caused by casual transmission of CA-MRSA strains. Although PVL gene is more frequently found in CA-MRSA strains, there are some reports of PVL gene recovered from HA-MRSA strains, but with a relatively lower prevalence [31, 37]. In this study, 17.9% of PVL-positive isolates were HA-MRSA. The presence of PVL gene among HA-MRSA strains and the interhospital spread of PVL encoding HA-MRSA strains support the need for system-wide implementation of patient safety and infection control initiatives.

Furthermore, PVL is carried by MSSA strains; thus, they share similar disease potential and epidemiological features with MRSA [49]. Based on most previous reports in Iran, the prevalence of PVL-positive MRSA strains is relatively higher than that of PVL-positive MSSA strains [38, 41, 50], in contrast to some European and UK countries where PVL-encoding MSSA strains are more common than PVL-positive MRSA strains [51, 52]. In agreement with most previous studies in Iran, about one third of the isolates (32.1%) were found to be PVL-positive MSSA in this study [42, 53]. However, in our previous study [54] and the study by Havaei et al. (2017) [55], the prevalence of PVL gene in MSSA strains was higher than in MRSA strains. Therefore, further in-depth studies are required to better understand the probable transfer and mobility of PVL gene among MSSA and MRSA clones.

PVL gene is highly conserved with four major variants (H1, H2, H3, R) identified based on the sequence variations at positions 527, 663, and 1396 [23]. H1 and H2 haplotype groups are more common in India and South Africa, whereas R variants are frequently found in the United States [23]. H variant (particularly the H2 group) has a broader geographical distribution and spreads within several CCs such as CC22, CC30, CC1, CC5, CC8, and CC121 [23, 55]. In this study, 53.6% of the isolates carried H2 variants, which were distributed among five CCs (CC22, CC30, CC1, and CC5). Moreover, 32.1% of R variants were allocated to three CCs including CC22, CC30, and CC8. There is very limited data about haplotype groups of *S. aureus* strains in Iran. In a recent study by Havaei et al. (2017), 56.6% of *S. aureus* isolates had H variants, and 43.3% carried R variants [55]. Their study revealed that both R and H variants were detected among *S. aureus* strains in Iran, consistent with the present study which showed that the *mec*A gene was present in 52.6% of the strains belonging to H variants. Additionally, some studies have reported that PVL-positive MRSA strains mainly belong to R variants, and only 5% of strains in H variants are *mec*A-positive [56]. In contrast, this may indicate controversial reports from different geographical locations.

It has been reported that PVL-positive MSSA and MRSA strains belong to diverse clones worldwide [49]. Although different clones were identified in this study, CC30 (31.6%) and CC22 (21%) were the predominant clones and PVL-positive S. *aureus* strains belonged to five CCs (CC30, CC22, CC8, CC1, CC5). The established four dominant MRSA clones consisted of CC30, CC22, CC1 and CC80. Two and three different SCC*mec* types were found in CC22 (III, IV) and CC30 (III, IV, V), respectively, and the majority of the strains (60%) in CC22 carried SCC*mec* type III. Moreover, most PVL-positive CA-MRSA strains belonged to CC30, whereas most PVL-positive HA-MRSA strains belonged to CC22. As previously reported, CC8, CC22, and CC30 are the dominant clones in Iran [28, 30, 42]. PVL-positive MRSA strains belonging to CC30 have been isolated in America, Europe, Asia, and the Southwest Pacific [57–59], while PVL-positive MRSA strains belonging to CC22 have been reported in England, Saudi Arabia, Germany, Ireland, Australia, and Nepal [58, 60, 61]. However, both CC22 and CC30 clones have been reported to be predominant in Asian countries [58, 62]. Based on the previous reports and also this study, it could be concluded that the most prevalent CA-MRSA clones in Asian countries including Iran are CC22 and CC30 clones [63, 64]. The predominance of CC22 and CC30 clones among PVL-positive MRSA strains in Iran is of great concern, as these clones appear to be highly transmissible with a propensity to spread worldwide. In the present study, 10.7% of *S. aureus* strains were related to CC8, and only one of them had SCC*mec* IV and was assigned to H2 variant. PVL-positive MRSA strains with CC8 and SCC*mec* IV have been previously reported in Iran [65]. CC8 is one of the most prevalent CCs worldwide and mainly contains nosocomial epidemic MRSA isolates [66]. The highest antibiotic resistance was observed in one PVL-positive HA-MRSA strain belonging to CC8. ST8 SCC*mec* type IV (USA300 clone) is the predominant clone in the United States but has also been reported in other countries including Anglophone, Pakistan, UK, and some of the Gulf States countries [67]. CC1 was another CC that was detected among PVL-positive strains in this study, accounting for 66.7% of R variant. According to the previous studies results, R variant of PVL is mainly found in CC1, CC8, and CC93 strains [68]. The present study results showed that R variant was harbored by 100% of PVL-positive MRSA strains primarily belonging to CC30, followed by CC1 and CC5, which were different from those CCs (CC8, CC1, and CC93) reported in previous studies [23, 55, 69]. This finding supports the findings of previous studies in Iran, in which CC30, CC22, CC8, and CC1 were detected as the predominant CCs among PVL-positive isolates [29].

In this study, the majority of the isolates (50%) belonged to *agr* type I. The *agr* locus belongs to the core variable genome and thus is linked to CCs. The *agr* typing results were consistent with the findings of previous studies in Iran and China [46, 47, 70]. In the current study, *agr* I and *agr* III were detected as the most common types and were linked to CC30, CC22, CC8, and CC1.

PFGE showed a high degree of genetic diversity among PVL-positive *S. aureus* clones and clustered them into A-C clusters and represented 24 PFGE pulsotypes. All R variants of PVL-positive MRSA strains belonged to cluster C, but H2 variants of these strains were distributed in all three clusters, supporting the hypothesis that H2 groups may display higher genetic diversity than other haplotype groups. This considerable diversity in PVL-positive MRSA strains could be explained by the possibility of isolating MRSA from different sources. Application of genotyping methods such as PFGE may provide a better interpretation of MRSA transmission sources and also help adopt well-intended infection prevention and control measures.

## Conclusion

To the best of our knowledge, this is the first comprehensive study investigating the molecular epidemiology and characteristics of PVL-encoding *S. aureus* clinical strains in Iran using several typing methods. The findings revealed i) a high prevalence of PVL-positive MRSA strains in Iran, ii) the majority of MRSA strains in the current study carried SCC*mec* IV, iii) both R and H variants were detected among *S. aureus* strains in Iran, iv) the *mec*A gene was present in 52.6% of the strains with H variants, v) CC8, CC22, and CC30 were found as the dominant clones among PVL-encoding *S. aureus* strains, vi) *agr* I and *agr* III were detected as the most common types and were linked to CC30, CC22, CC8, and CC1. The most important limitation of the present work was the relatively small sample size, because of low prevalence of MRSA strains. Taken together, the present study results may contribute to the understanding of the molecular epidemiology and evolution of PVL-positive *S. aureus* in Iran.

## Data Availability

The partial nucleotide sequences of *LukSF-PV* genes of 28 strains generated and/or analyzed during the current study are available in the GenBank/NCBI database (https://www.ncbi.nlm.nih.gov/genbank) under the following accession numbers: MT468493-MT468548.
